# Cellular Defense and Sensory Cell Survival Require Distinct Functions of *ebi* in *Drosophila*

**DOI:** 10.1371/journal.pone.0141457

**Published:** 2015-11-02

**Authors:** Young-Mi Lim, Yoshimasa Yagi, Leo Tsuda

**Affiliations:** 1 Animal Models of Aging Project Team, Center for Development of Advanced Medicine for Dementia (CAMD), National Center for Geriatrics and Gerontology (NCGG), Obu, Aichi, Japan; 2 Division of Biological Science, Graduate School of Science, Nagoya University, Nagoya, Aichi, 464–8602, Japan; Oxford Brookes University, UNITED KINGDOM

## Abstract

The innate immune response and stress-induced apoptosis are well-established signaling pathways related to cellular defense. NF-κB and AP-1 are redox-sensitive transcription factors that play important roles in those pathways. Here we show that Ebi, a *Drosophila* homolog of the mammalian co-repressor molecule transducin β-like 1 (TBL1), variously regulates the expression of specific genes that are targets of redox-sensitive transcription factors. In response to different stimuli, Ebi activated gene expression to support the acute immune response in fat bodies, whereas Ebi repressed genes that are involved in apoptosis in photoreceptor cells. Thus, Ebi seems to act as a regulatory switch for genes that are activated or repressed in response to different external stimuli. Our results offer clear *in vivo* evidence that the Ebi-containing co-repressor complex acts in a distinct manner to regulate transcription that is required for modulating the output of various processes during *Drosophila* development.

## Introduction

The innate immune response is a cellular defense system that protects animals from threats such as external infections and is required for maintenance of normal physiology [1]. The innate immune system seems to be evolutionarily conserved; in fact, *Drosophila melanogaster* and humans share conserved signaling machinery including receptors, signaling mediators, and transcription factors that govern the response [2]. In particular, nuclear factor-kappa B (NF-κB) plays a key role in regulating transcriptional events of the response [2, 3]. *Drosophila* contains three types of NF-κB-like transcription factors, namely Dorsal, Dorsal-related immunity factor, and Relish (Rel) [2]. A large body of evidence suggests that NF-κB works together with several distinct transcription factors such as the antioxidant response element—binding protein activator protein-1 (AP-1) [4, 5]. NF-κB and AP-1, which are called redox-sensitive transcription factors, participate not only in the innate immune response but also in inflammation, cancer formation, and stress-induced apoptotic processes in cells [6, 7].

AP-1 and NF-κB can act as both transcriptional activators and repressors of specific target genes [8, 9, 10, 11]. A growing number of studies have shown that many kinds of cofactors are involved in these events [12, 13]. Among the cofactors, co-repressor complexes, such as nuclear receptor co-repressor (N-CoR) and the silencing mediator for retinoid and thyroid hormone receptor (SMRT) complex, appear to play important roles in reactive oxygen species—induced apoptotic signaling pathways and the innate immune response, which are both regulated by AP-1 and NF-κB [14, 15, 16, 17]. Moreover, the N-CoR/SMRT repressor complex regulates the expression of many types of genes by associating with transducin β-like protein 1 (TBL1) and TBL1-related protein (TBLR1), two highly related F-box/WD-40–containing factors [18, 19, 20, 21]. Notably, TBL1 and TBLR1 act as exchange factors that facilitate the addition and/or extraction of factors of the transcriptional repressor complex to yield a transcriptionally active complex at the genomic target sites of various nuclear hormone receptors [16, 22]. However, Yoon *et al*. failed to observe transcriptional activation mediated by TBL1/TBLR1, and therefore the precise activities of these factors as transcriptional activators remain enigmatic [23].

Ebi, the *Drosophila* homolog of TBL1/TBLR1, is also a transcriptional co-repressor when present in a complex with SMRTER, the *Drosophila* counterpart of N-CoR/SMRT [24, 25, 26]. A recent study showed that Ebi can associate with AP-1 and that the repressor activity of Ebi is required for long-term survival of sensory photoreceptor cells [27]. AP-1/Ebi activity is required for suppression of pro-apoptotic genes (PAGs) such as *hid*, *grim*, *reaper*, and *sickle*. Loss of function of *ebi* causes late-onset photoreceptor cell degeneration that is AP-1 dependent [27].

Herein we analyzed the function of Ebi in the cellular defense response using RNA interference (RNAi)-mediated knockdown. Our work clearly shows that Ebi acts as a positive regulator for the transcriptional regulation mediated by Rel (NF-κB) and AP-1 in fat bodies. Conversely, Ebi represses Rel and AP-1 target genes in other organs including the eye disc. These results indicate that Ebi has two distinct functions with respect to regulating the expression of Rel and AP-1 target genes. The distinct function of Ebi might be required for maintaining appropriate activation of specific pathways in different tissues. Hence, Ebi seems to act as a molecular switch that modulates the output of genes that are regulated by redox-sensitive transcription factors.

## Results

### *ebi* is required for the innate immune response in the presence of bacterial infection

**Fig 1:**
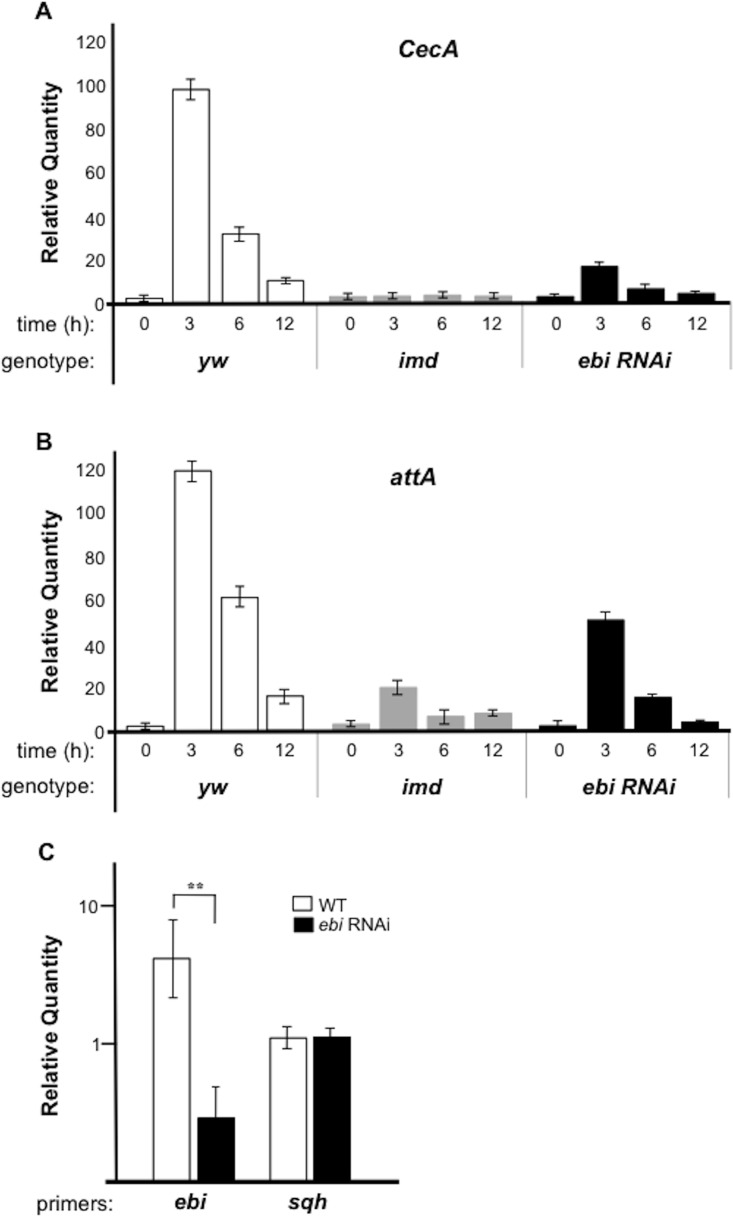
*ebi* is required for Rel target gene expression during bacterial infection. qPCR analysis of mRNA from larvae of *yw* (n = 30, each time point), *Cg-Gal4>ebi RNAi* (n = 30, each time point), or *imd* mutant larvae (n = 30, each time point). The experiment was performed two times. Expression of *CecA* (A) and *attA* (B) was assessed at different times after bacterial challenge (0, 3, 6, 12 h). The experiment was performed three times. (C) qPCR analysis of mRNA from wild-type (WT) (n = 25) or after *Cg-Gal4>ebi RNAi* (n = 28). *Sqh* (*spaghetti squash)* was used as a control for mRNA level. The data represent the mean ± SD. **p < 0.01, *vs*. mock with *ebi* RNAi. The data was plotted by a log scale.

Our previous study revealed that Ebi associates with and regulates the transcriptional activity of AP-1 [27]. It has been shown that AP-1–mediated transcriptional activity regulates the innate immune response [28, 29]. Thus, we investigated whether Ebi is also involved in the cellular defense response in *Drosophila*. In the *Drosophila* innate immune response, the expression of anti-microbial peptide genes (AMPs) is regulated by Rel together with AP-1 [29]. Therefore, we examined the function of Ebi in the regulation of AMP expression. In the presence of a Gram-negative bacterial challenge, the expression of AMPs such as *Cecropin A* (*CecA*) and *attacinA* (*attA*) was induced in whole larvae (Fig 1A and 1B; S1 Fig). It has been shown that increased levels of AMP products during this period is due mainly to AMP expression in fat bodies [2]. We thus speculated that Ebi might be involved in modulating the activities of AP-1 and NF-κB. Because the *Drosophila* immune deficiency (*imd*) signaling pathway regulates both AP-1 and NF-κB activities, *imd* is an ideal tool to analyze the functional relationship between Ebi and redox-sensitive transcription factors such as NF-κB and AP-1 [30]. Mutation of *imd* prohibits AMP induction during bacterial infection [30] (Fig 1A and 1B). Using fat bodies and the hemocyte *Gal4* line (*Cg-Gal4*), we introduced double-stranded RNA (dsRNA) targeting *ebi* (*ebi* ^*HMS01390*^, for the purpose of RNAi) into fat bodies and observed >70% reduction in *ebi* mRNA level in this organ (Fig 1C). Under this condition, we found that reduced Ebi activity in fat bodies led to decreased expression of AMPs, suggesting that Ebi positively regulates Rel target genes (Fig 1A and 1B).

**Fig 2:**
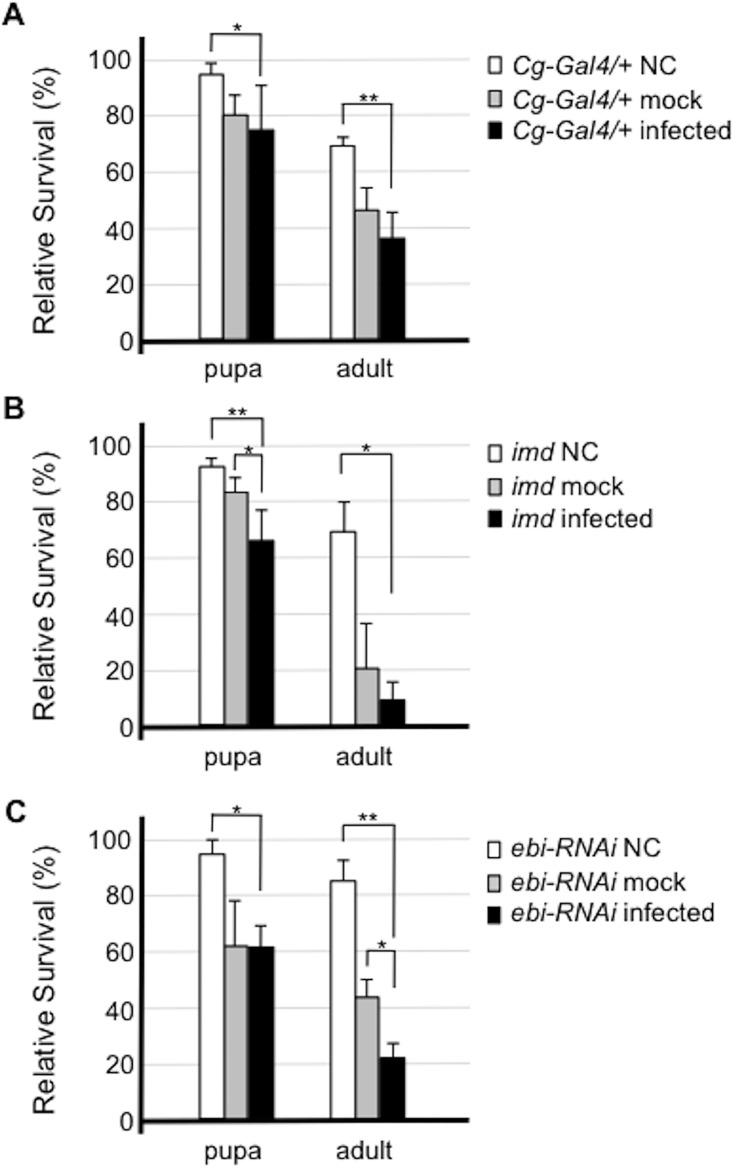
Ebi is involved in the cellular defense response against bacterial infection. Susceptibility to *Enterobacter cloacae* in flies of each genotype was assessed. Flies were infected with *E*. *cloacae* by pricking, and the percentage of surviving flies 2 days after infection was calculated. Error bars denote standard error. The experiment was performed four times. Both *Cg-Gal4/+* (control) flies (A) and *Imd* mutant flies (B) showed increased sensitivity to bacterial challenge. *Cg-Gal4>ebi RNAi*, in which *ebi* expression was inhibited, showed enhanced lethality to bacterial challenge (C). Data represent the mean ± SD. *p < 0.05; **p < 0.01. NC: not challenged (not pricked); mock: mock infection (pricked with a clean needle); infected: infected by pricking the insect with a pin inoculated with bacteria.

Our data thus far indicated that Ebi may mediate cellular defense signaling against Gram-negative bacterial infection. It has been shown that the innate immune system in *Drosophila* supports the survival of each animal upon bacterial infection [2]. Thus, we assessed the survival of both larvae and adults after bacterial infection. *Imd* mutants had a markedly decreased survival rate at both the pupal and adult stages upon infection (Fig 2B, compare to Fig 2A). Larvae in which *ebi* RNAi was introduced in fat bodies also had significantly reduced survival of adult flies upon bacterial challenge (Fig 2C). These results suggested that Ebi activity in fat body cells is required for the innate immune response in the presence of infectious stimulation.

### Ebi is a transcriptional activator in the fat bodies

**Fig 3:**
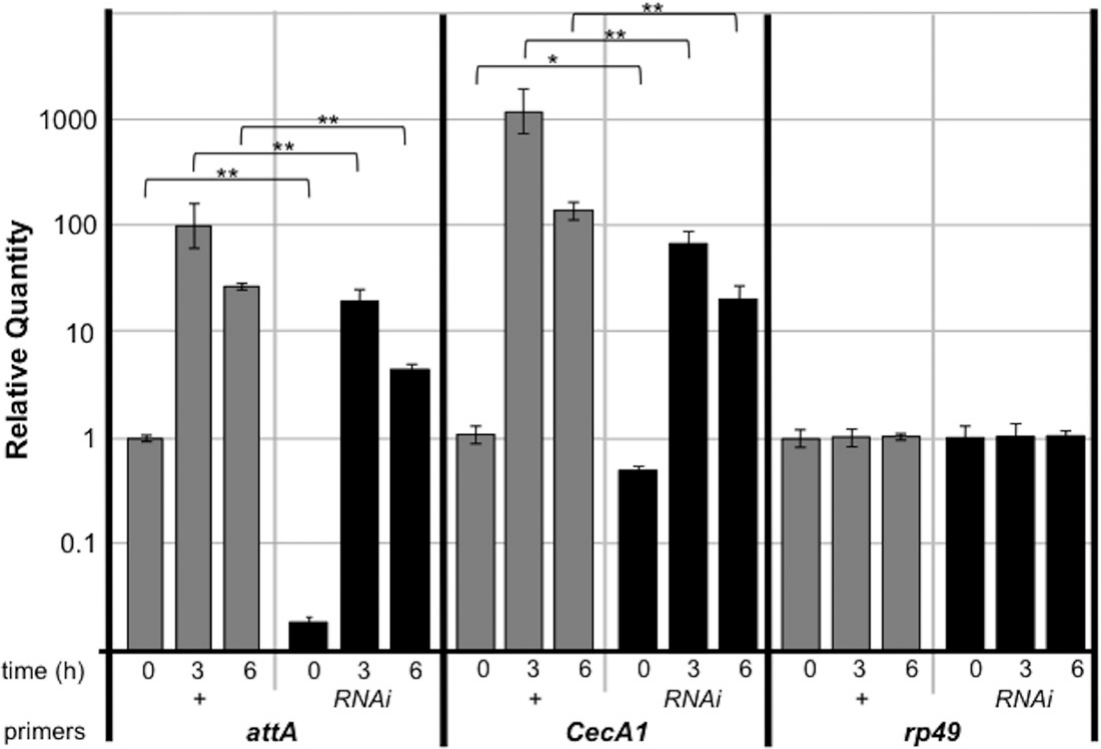
Ebi positively regulates Rel target genes in fat bodies. Real-time qPCR analysis of mRNA from fat bodies isolated from larvae of *Cg-Gal4/+* (+) or *Cg-Gal4>ebi RNAi* (RNAi) (n = 30 for each time point). Time: time elapsed after bacterial infection Specific primers for each gene were analyzed. Data represent the mean ± SD. *p < 0.05; **p < 0.01. The experiment was performed three times. The data was plotted by a log scale.

We found that treatment of fat bodies with *ebi*-specific RNAi decreased the expression of AMPs as assessed with mRNA isolated from whole larvae. To investigate whether the regulation of Ebi against AMPs are cell autonomous, we analyzed AMP expression to be limited in fat bodies. We checked up the reduction in expression of AMPs when Ebi was inhibited in fat bodies (Fig 3). We noticed that *attA* and *CecA1* were expressed at their basal levels during *ebi* RNAi, suggesting that cell-autonomous Ebi function is necessary for AMP expression. We confirmed these results using an independent *ebi* RNAi line (*ebi* ^*GLC01413*^), which indicated that the results were probably not a consequence of off-target effects (S2 Fig).

**Fig 4:**
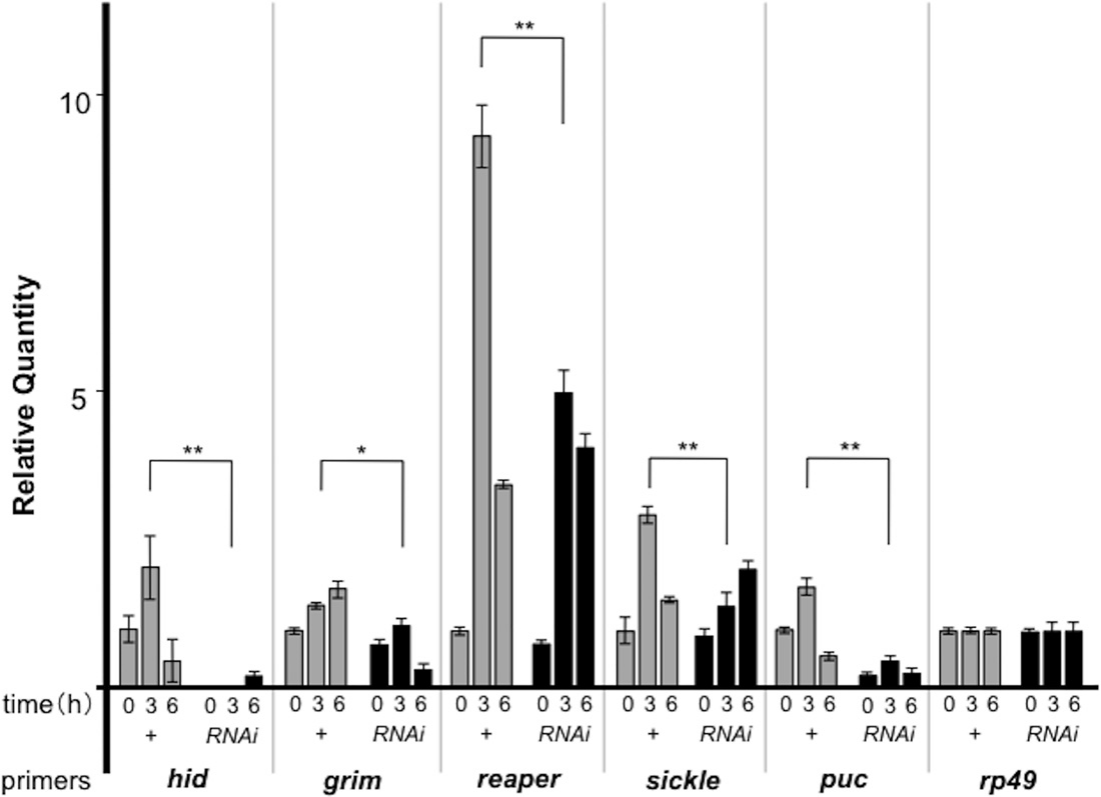
Ebi positively regulates AP-1 target genes in fat bodies. qPCR analysis of mRNA from larvae of *yw* (+) or *Cg-Gal4>ebi RNAi* (RNAi) (n = 30, each time point). Time: time elapsed after bacterial infection. *hid*: *head involution defective* (also know as *W*); *puc*: *puckerd*. *Rp49* was used as a control. Data represent the mean ± SD. *p < 0.05; **p < 0.01. The experiment was performed three times.

We previously reported that Ebi represses PAGs, many of which are targets of AP-1, in photoreceptor cells and S2 cells, and that loss of *ebi* function results in upregulation of PAGs [27]. We thus monitored the expression of *hid*, *grim*, *reaper*, and *sickle* before and after bacterial challenge. Although the basal expression of these genes was relatively low, the expression of all the PAGs did not increase with *ebi* RNAi, and rather it seemed that expression was reduced when *ebi* was inhibited (Fig 4). We also confirmed that expression of *puckered* (*puc*), which is another AP-1 target gene, was also inhibited by *ebi* RNAi (Fig 4) [31]. These results indicated that, in fat bodies, Ebi itself functions as an activator rather than a repressor.

### Ebi downregulates Rel target genes in non—fat body tissues

**Fig 5:**
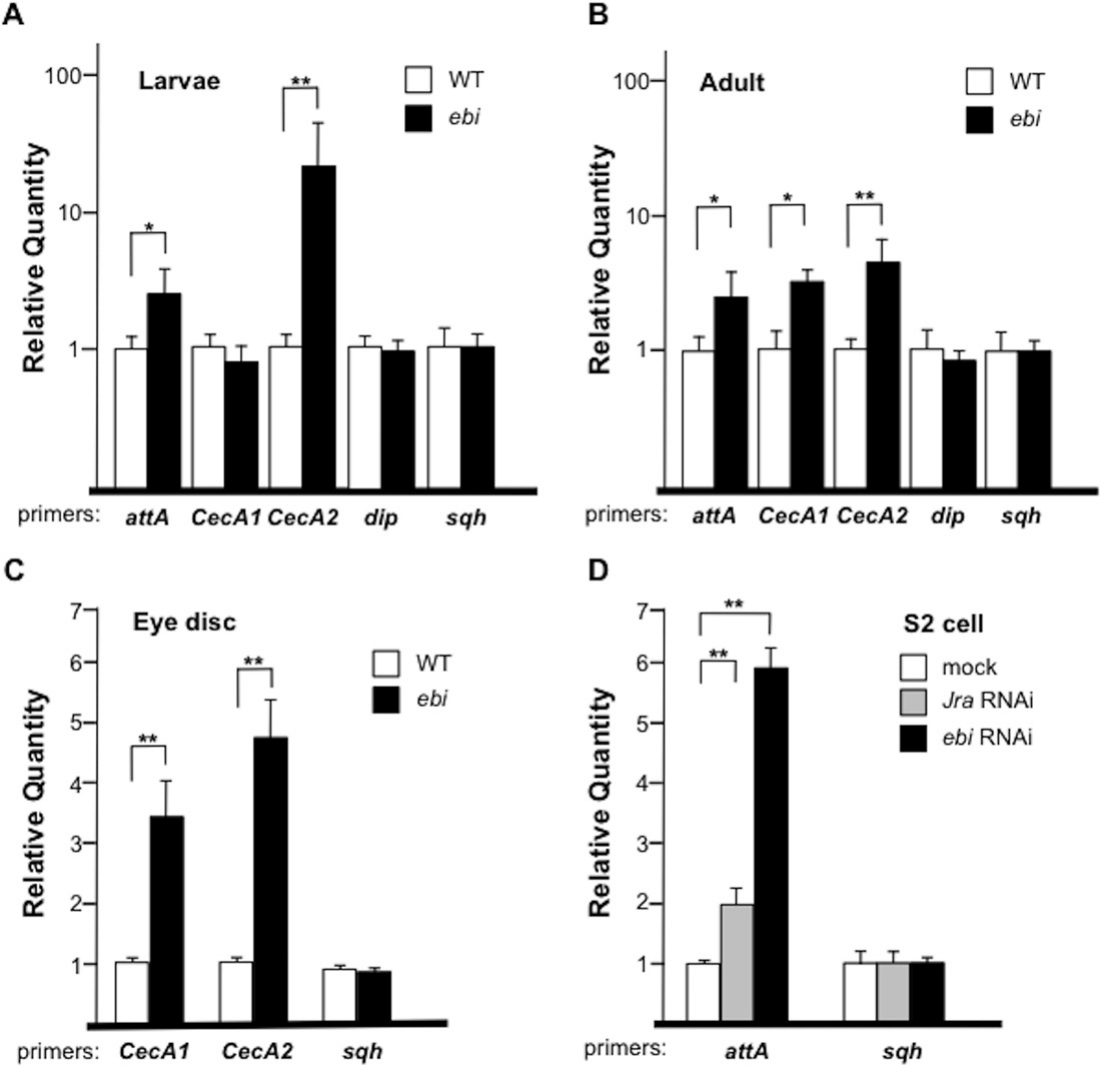
Ebi and AP-1 antagonize *IMD* signaling. (A–D) qPCR analysis of mRNA from wild-type (WT) or *ebi* mutant (*ebi* ^*P*^ */ebi* ^*4*^) first-instar larvae (n = 36 and 25, respectively) (A), from WT or *ebi* mutant escaper (*ebi* ^*7*^ */ebi* ^*90*^) adult flies (n = 30 and 21, respectively) 1 day after eclosion (B), from WT or *ebi* mutant escaper (*ebi* ^*11*^ */ebi* ^*4*^) third-instar larvae eye-antennal discs (n = 80 and 65, respectively) (C), and from S2 cells treated with mock dsRNA, dsRNA against *Jra* (RNAi), or *ebi* RNAi (D). *attA*: *attacinA*; *sqh*: *spaghetti squash*. Data represent the mean ± SD. *p < 0.05; **p < 0.01. (A) and (B) were platted by a log scale.

To examine whether the Ebi-mediated upregulation of AMP expression is fat-body specific, we assessed the effect of Ebi on AMP expression in non—fat body tissues. First, we monitored AMP expression in *ebi* mutant larvae and adult flies. For the *ebi* mutant analysis, we used several different mutant strains; the combination of *ebi* ^*P*^ and *ebi* ^*4*^ has been shown to yield a severe loss-of-function phenotype that results in lethality at approximately the first or second instar larval stage, and the combination of strains *ebi* ^*7*^ and *ebi* ^*90*^ yields adult escapers [24, 26]. In this experiment, we found that some, if not all, AMPs such as *CecA* and *attA* were ectopically induced in *ebi* mutant larvae and adult flies in the absence of bacterial infection (Fig 5A and 5B). To clarify the tissue specificity of Ebi activity with respect to Rel target genes, we assessed *CecA* expression in eye-antenna discs. In this case we used the mutant combination *ebi* ^*11*^ and *ebi* ^*4*^, which is lethal at the pupal stage [26]. We observed increased expression of *CecA1* and *CecA2* in *ebi* mutant eye-antenna discs (Fig 5C). To assess the cell autonomy of the effect of Ebi in the negative regulation of AMP expression, we tested the role of Ebi in the expression of endogenous *attA* in S2 cells. As with a previous study that showed that *attA* expression is repressed by AP-1 [29], RNAi directed towards *Jra* (*Drosophila* c-jun) caused a 2-fold increase in *attA* expression (Fig 5D). We also found that reduced *ebi* expression greatly increased *attA* expression (Fig 5D). These results supported the idea that Ebi may play a role as a repressor of AMP transcription in non—fat body tissues such as eye-antenna discs.

### Ebi regulates AMP expression through the promoter region

**Fig 6:**
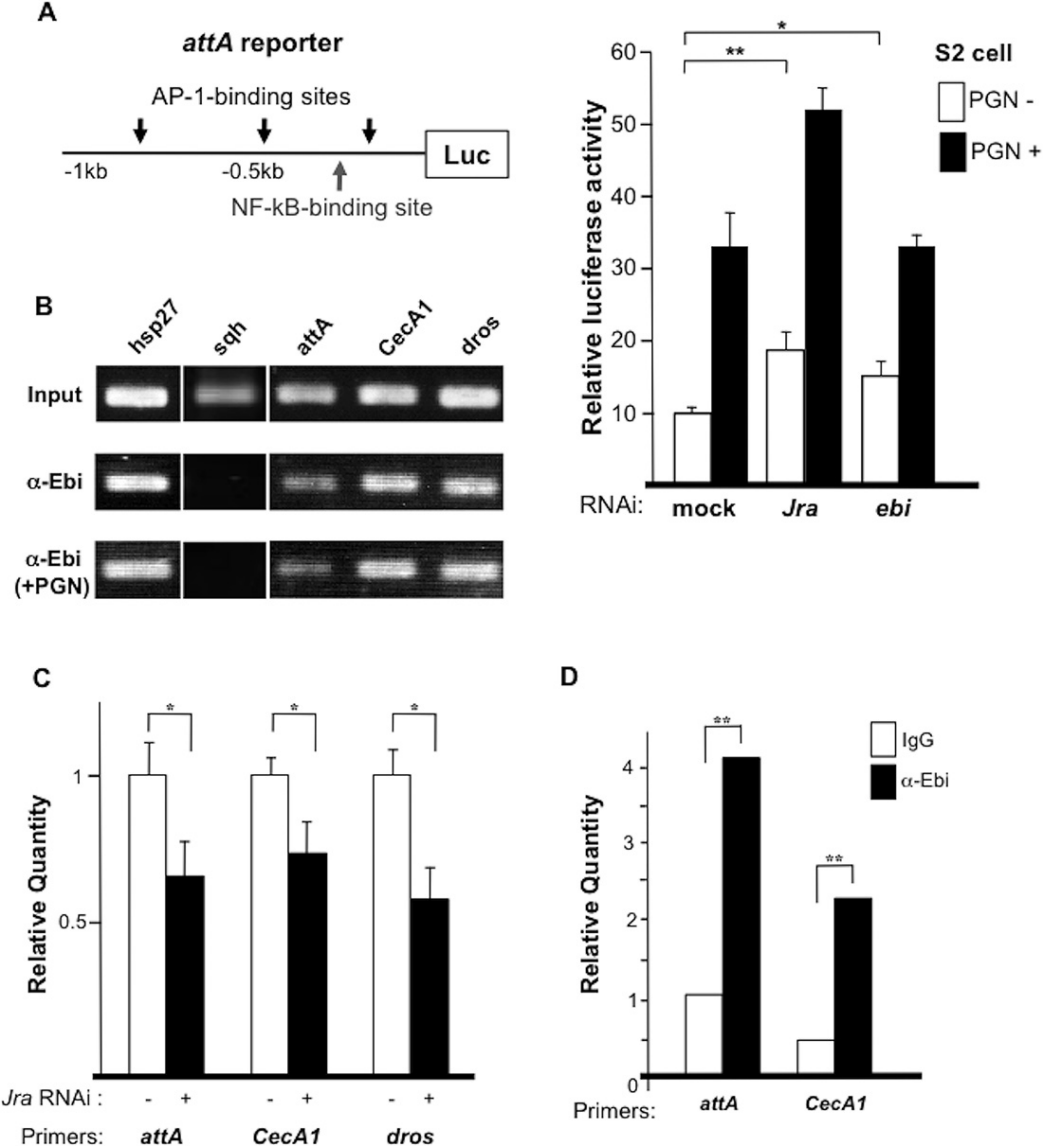
Ebi directly regulates the expression of AMPs. (A) Left: Reporter analysis using the *attA* promoter region; right: dsRNA-mediated knockdown of *Jra* or *ebi* in the absence or presence of peptidoglycan (PGN). Data represent the mean ± SD. *p < 0.05; **p < 0.01. The experiment was performed three times. (B) ChIP analysis using different AMPs as a probe. The positive control was *hsp27*. The experiment was performed two times. (C) ChIP-qPCR results obtained with primers specific to the promoter regions of AMPs with anti-Ebi in the absence (white bars) or presence (black bars) of *Jra*-specific dsRNA in S2 cells. Amplification was normalized to the control without dsRNA treatment. In all cases, the enrichment of each promoter region was inhibited by dsRNA against *Jra*. Data represent the mean ± SD. *p < 0.05; **p < 0.01. The experiment was performed three times. (D) ChIP-qPCR results obtained with primers specific to the promoter regions of AMPs (*attA* and *CecA1*) with anti-Ebi (black bar) or IgG (white bar) in fat bodies (n = 100). Amplification was normalized to the internal control and calculated for each input. **p < 0.01.

Next, we performed a reporter gene assay using an *attA* reporter containing binding sites for NF-κB and AP-1 upstream of the luciferase gene (Fig 6A) [32]. Peptidoglycan stimulated IMD signaling and *attA* expression by more than 3-fold (Fig 6A) [33]. The basal and peptidoglycan-induced activities of the reporter were increased following treatment with *Jra* dsRNA (i.e., RNAi) (Fig 6A) [29]. Although *ebi* downregulation did not enhance the reporter activity upon stimulation with peptidoglycan (Fig 6A, right), RNAi-induced reduction in *ebi* expression induced a small but significant elevation in the basal reporter activity. These results suggested that Ebi represses AMP expression mainly through AP-1.

### Direct recruitment of Ebi to the promoter region of AMPs

To investigate how Ebi regulates AMP expression at the molecular level in S2 cells, we performed chromatin immunoprecipitation (ChIP). Ebi was found to be associated with the promoter regions of *attA*, *CecA1*, *and dros* (Fig 6B). Because many AMP promoter regions contain binding sites for both AP-1 and NF-κB, we tested whether Ebi recruitment to promoters is AP-1 dependent [32]. We introduced dsRNA against *Jra* and found that recruitment of Ebi to the promoter regions of AMPs was decreased (Fig 6C), which is consistent with a previous report showing that Ebi associates with AP-1 and represses AP-1 target genes [27]. The observation that stimulation with peptidoglycan did not change the recruitment of Ebi to the promoter regions of those genes (Fig 6B) suggested that Ebi is continuously recruited to AMP promoter regions via AP-1. To elucidate the *in vivo* function of Ebi, we performed ChIP using fat-body extract, which revealed that Ebi associated with the *attA*, *CecA1* and *hid* promoter regions in fat bodies (Fig 6D; S3 Fig). These results suggested that Ebi is directly involved in the transcriptional regulation of AMPs and PAGs in fat bodies.

## Discussion

Ebi plays distinct roles in the expression of target genes in different tissues [34]. When flies are challenged by external stimuli such as bacterial infection, cells in the fat body require high expression of AMPs to mount an adequate defense response [3]. Under such conditions, the co-repressor system may be an obstacle for the organism. Therefore, Ebi-containing co-repressors may be converted to activator complexes to allow efficient expression of AMPs. In non—fat bodies such as photoreceptor cells, high levels of AMP expression are not required, and cells must be protected from apoptotic induction during stress signaling. Under these conditions, Ebi seems to act as part of a co-repressor complex to repress any excess expression of PAGs.

It has been shown that most of the promoter regions of AMPs contain consensus sequences for binding NF-κB and AP-1 [32]. There seems to be a balance for the utilization of these two transcription factors. In cultured S2 cells, AP-1 acts as a negative regulator of AMP transcription, whereas AP-1 is required for AMP activation in fat bodies. In support of this notion, *Jra* knockdown did not impair AMP expression in S2 cells (Fig 6A), whereas it impairs PAGs expression in S2 cells [27].

Actually, the diverse function of Ebi against target gene expression seems to be evolutionarily conserved. TBL1 is involved in transcriptional activation as well as repression [16]. TBL1/TBLR1 seems to act as a specific adaptor molecule that mediates the exchange of co-repressors for co-activators [16]. This TBL1/TBLR1-based molecular switch may contribute to the efficient response to external signaling [16].

Protein degradation may contribute to the exchange activity of TBL1/TBLR1 [16]. TBL1/TBLR1 seems to act as a specific adaptor molecule for recruitment of the ubiquitin-conjugating 19S proteasome, which regulates the stability of the regulatory machinery for transcription factors, to promoter regions of target genes [16]. Intriguingly, Ebi also seems to mediate specific protein degradation during development [24, 35, 36]. Therefore, we cannot exclude the possibility that Ebi contributes indirectly to the regulation of AMP transcription via protein degradation. Hence, we predict that Ebi, like TBL1/TBLR1 in mammals, may be involved in switching transcriptional repression to activation by regulating the turnover of particular transcriptional cofactors.

External signaling pathways are thought to interact with co-repressors and co-activators [12]. Notably, Ebi has been shown to act under several distinct signaling pathways, such as those governed by *EGF receptor*, *Notch*, or *wingless* [24, 25, 37].

A recent study indicated that the opposing functions of the TBL1/TBLR1 complex are regulated by the Toll-like receptor signaling pathway through phosphorylation of N-CoR [38]. This suggests that extracellular signaling is closely related to the regulation of the opposing activities of TBL1/Ebi family molecules in transcriptional regulation. Thus, we expect that cellular signaling, such as that mediated by the Toll pathway, may contribute to Ebi activity as a transcriptional activator of AMPs in fat bodies.

## Materials and Methods

### *Drosophila* stocks

The following stocks were used in this study: Oregon-R as a wild type, *ebi* ^*4*^, *ebi* ^*90*^, *ebi* ^*P*^, *ebi* ^*p7*^, and *ebi* ^*11*^ [24, 25]; *y* ^*1*^ *w* ^*1118*^; *imd* ^*1*^ [39]; *GMR-Gal4* [40]; *UAS-imd*, *UAS-RelN*, *Cg-Gal4* [41], *ebi* ^*HMS01390*^ (*ebi RNAi*) and *ebi* ^*GLC01413*^ (*ebi RNAi*) was obtained from the Bloomington Drosophila Stock Center, USA. *egr* ^*GS1226*^ was obtained from the Kyoto Stock Center, Japan.

### Bacterial infection of larvae

The bacterial infection assay was performed as described [41]. Briefly, wandering third instar larvae were washed with distilled water before infection. Bacterial infection was carried out by puncturing larvae with a needle that was previously dipped in a solution of *Enterobacter cloacae*. After infection, the larvae were placed on wet filter paper inside a moist chamber and collected into tubes 0, 3, 6, or 12 h after infection and stored at −80°C until RNA extraction.

For the survival assay, wandering third instar larvae (n = 40, each) were infected and placed on wet filter paper inside a moist chamber for 1 h after infection. Larvae that did not die because of injury were moved to a culture tube, and surviving pupae or adult flies were counted.

### Peptidoglycan stimulation in S2 cells

Lipopolysaccharide (Sigma) was added to the medium of S2 cells (1 × 10^6^ cells) to a final concentration of 1 μg/ml. After 1.5 h, cells were collected and mRNA isolation or ChIP analysis performed.

### Primer information

All the primer information is described in S1 Methods.

### Real-time quantitative PCR (qPCR)

Most qPCR procedures were performed as described [27]. Briefly, RNA was extracted using an RNA purification kit (QIAGEN), and each cDNA was synthesized with Primescript RT reagent (TAKARA). The mRNA level was quantified using a Thermal Cycler Dice Real Time System with SYBR Premix Ex Taq (TAKARA). Data were normalized to *rp49* or *sqh* mRNA. The thermal cycling parameters were 40 cycles of 95°C for 10 s and 60°C for 30 s.

Each time sample was duplicated, and at least two independent experiments were performed for each data analysis.

### Luciferase assay

A reporter construct for *attA-luc* was obtained from Dr. Jean-Luc Imler. The reporter constructs were transfected into S2 cells (1 × 10^6^ cells) using Effectene Reagent (QIAGEN), together with *pActin-RL* in the presence or absence of dsRNA. After 48 h, the cells were lysed, and firefly luciferase activity was analyzed with the dual luciferase reporter assay system (Promega). Luciferase activity in each sample was normalized to *Renilla* luciferase activity.

### ChIP analysis

S2 cells (1 × 10^7^) were collected in phosphate-buffered saline, fixed with 1% formaldehyde for 15 min at room temperature, and subjected to ChIP as described [42]. Cross-linked adducts were resuspended and sonicated, resulting in DNA fragments of 500–1000 bp. Immunoprecipitation was performed using an rabbit polyclonal antibody against Ebi [24]. Protein-bound, immunoprecipitated DNA was dissolved in Tris/EDTA buffer (pH 7.8) and incubated at 65°C for 6 h. Digestion buffer (10 mM Tris-HCl, 100 mM NaCl, 25 mM EDTA, pH 8.0) was added to the sample and incubated for 1 h at 45°C with 0.1 mg/ml proteinase K (Sigma). DNA was purified using the PureLink Plasmid purification kit (Invitrogen) and used as a template for qPCR. The primer set for *hsp27*, which is a target site for ecdysone receptor (EcR), was used as a positive control [26]. Oligonucleotides for ChIP analysis, real-time qPCR, and dsRNA for ChIP analysis are described in the supplemental information.

### Acridine orange staining

Acridine orange staining was performed as described [40]. Eye-antennal discs were dissected in phosphate-buffered saline and incubated with 1.6μM acridine orange solution in *Drosophila* Ringer [43] and then mounted in Vectashield (Vector Laboratories).

### Histochemistry

Sectioning of eyes and epon embedding were carried out as described [24]. Briefly, dissected eyes were fixed in 2.5% glutaraldehyde, dehydrated, and embedded in epon plastic. Thin sections were stained with toluidine blue for light microscopy.

Additional Materials and Methods are described in S1 Methods.

## Supporting Information

S1 Fig *sqh* gene expression during bacterial infection. qPCR analysis of mRNA from larvae of *yw*, *Cg-Gal4>ebi RNAi*, or *imd* mutant larvae. *sqh* expression was observed at different times after bacterial challenge (0, 3, 6, 12 h). Data represent the mean ± SD.
(TIF)

S2 Fig Ebi positively regulates Rel target genes in fat bodies. qPCR analysis of mRNA from larvae of *Cg-Gal4/+* (+) or *Cg-Gal4>ebi RNAi* (*ebi* ^*GLC01413*^) (n = 30 for each). The word “time” implies the time after bacteria infection. Specific primers for each gene were analyzed. Data represent the mean ± SD. *p < 0.05; **p < 0.01. n = 4. The experiment was performed three times.
(TIF)

S3 Fig Ebi directly regulates the expression of PAGs in fat bodies. ChIP-qPCR results obtained with primers specific for the promoter regions of PAGs (*hid*) using anti-Ebi (black bar) or IgG (control, white bar) in fat bodies (n = 100). The amplified products were adjusted according to the internal control, and the net amount of each product was calculated for each input. Data represent the mean ± SD. **p < 0.01. The experiment was performed two times.
(TIF)

S1 Methods Oligonucleotides for ChIP analysis, real-time PCR, and dsRNA for ChIP. (DOC)
