# Supplementary figures and images for: Cellular Defense and Sensory Cell Survival Require Distinct Functions of ebi in Drosophila
Source: PLoS One. 2015 Nov 2;10(11):e0141457. doi: 10.1371/journal.pone.0141457 (PMC4629896; doi:10.1371/journal.pone.0141457)

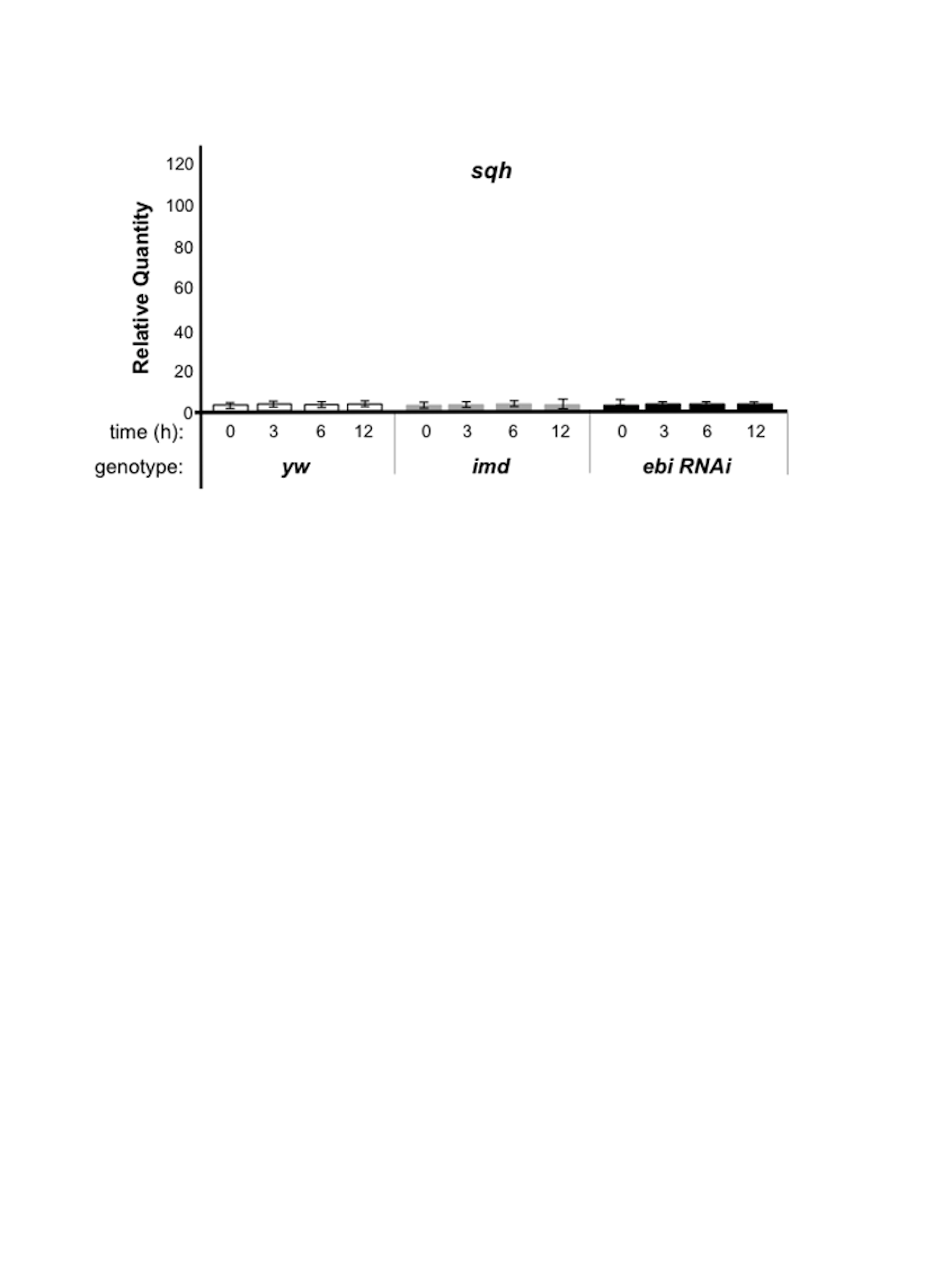

Supplement: S1 Fig — qPCR analysis of mRNA from larvae of yw, Cg-Gal4>ebi RNAi, or imd mutant larvae. sqh expression was observed at different times after bacterial challenge (0, 3, 6, 12 h). Data represent the mean ± SD. (TIF) [file pone.0141457.s001.tif]

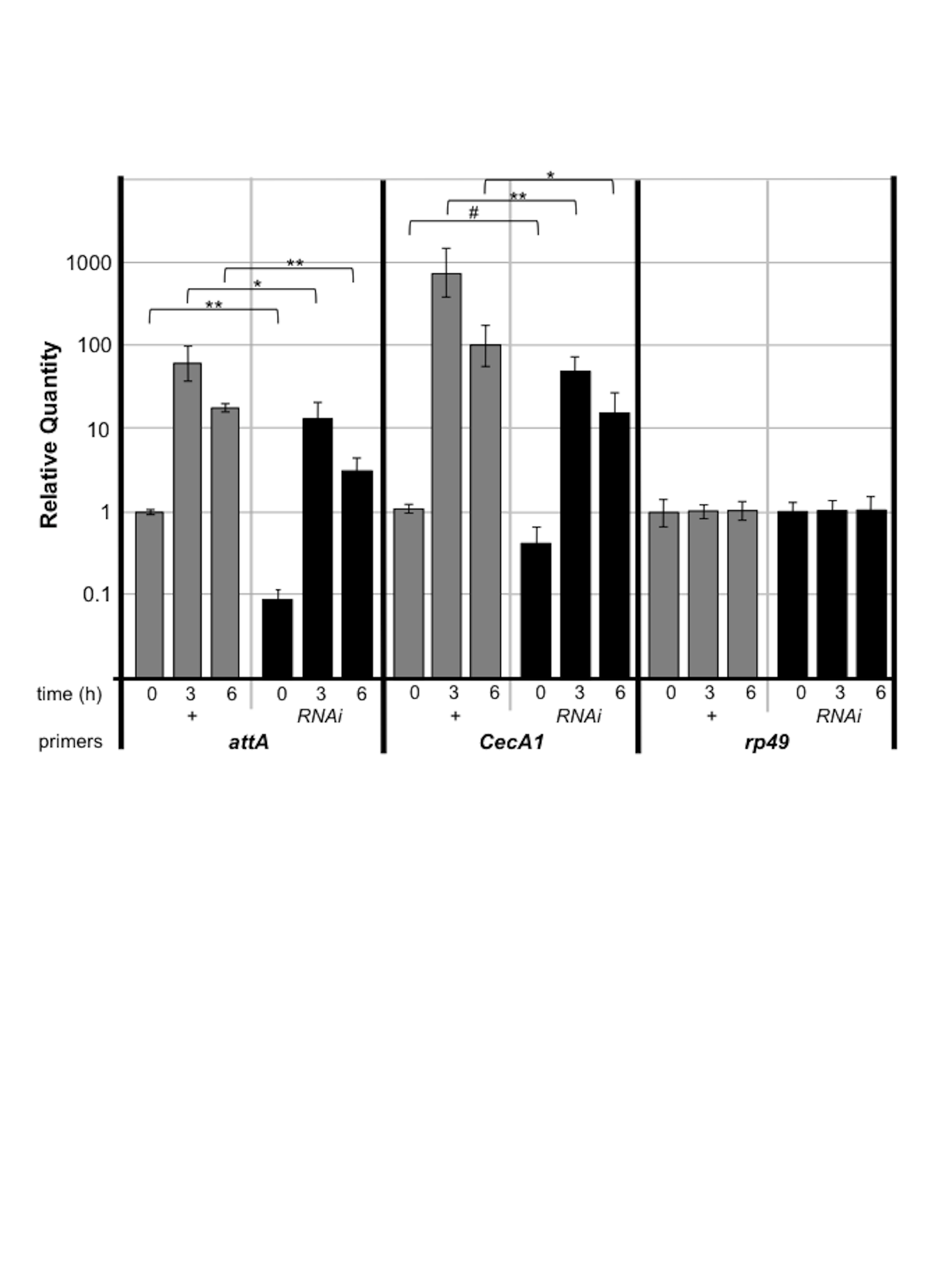

Supplement: S2 Fig — qPCR analysis of mRNA from larvae of Cg-Gal4/+ (+) or Cg-Gal4>ebi RNAi (ebi GLC01413) (n = 30 for each). The word “time” implies the time after bacteria infection. Specific primers for each gene were analyzed. Data represent the mean ± SD. *p < 0.05; **p < 0.01. n = 4. The experiment was performed three times. (TIF) [file pone.0141457.s002.tif]

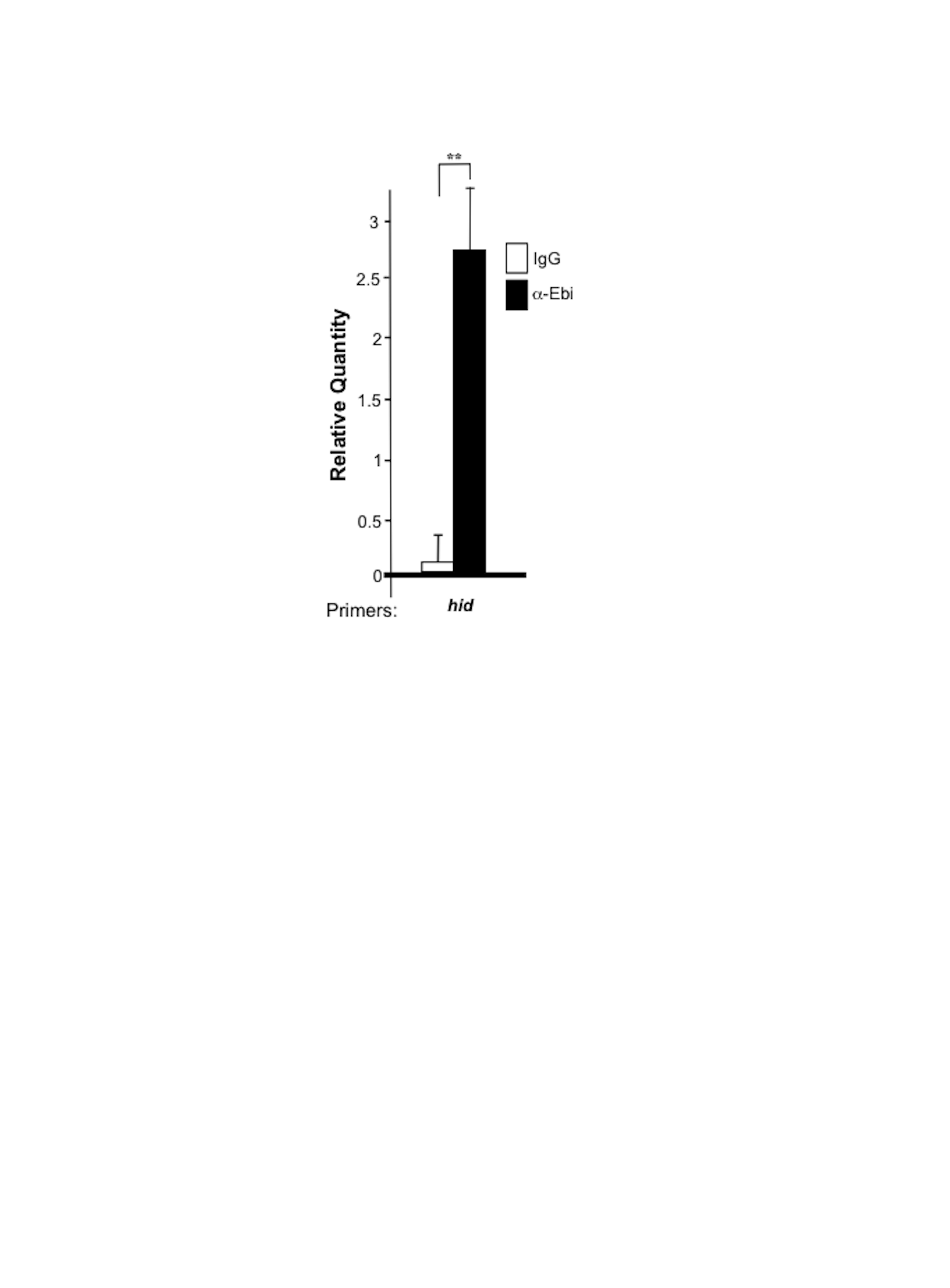

Supplement: S3 Fig — ChIP-qPCR results obtained with primers specific for the promoter regions of PAGs (hid) using anti-Ebi (black bar) or IgG (control, white bar) in fat bodies (n = 100). The amplified products were adjusted according to the internal control, and the net amount of each product was calculated for each input. Data represent the mean ± SD. **p < 0.01. The experiment was performed two times. (TIF) [file pone.0141457.s003.tif]
