# Supplementary material for: Cellular Defense and Sensory Cell Survival Require Distinct Functions of ebi in Drosophila
Source: PLoS One. 2015 Nov 2;10(11):e0141457. doi: 10.1371/journal.pone.0141457 (PMC4629896; doi:10.1371/journal.pone.0141457)
Supplement: S1 Methods — (DOC) [file pone.0141457.s004.doc]

**S1 Methods**

**Oligonucleotides for ChIP analysis, real-time PCR, and dsRNA**

For ChIP analysis

*attA* (ChIP):

Sense: 5′-GCGGGGAATTTCGCTTTG AT-3′

Antisense: 5′-TTGCTGAACTGGATTGCTGG-3′

*CecA1* (ChIP):

Sense: 5′-AATCCCCGATTGTTCCCTAG-3′

Antisense: 5′-CTGCGATACAAAAGGCGAGA-3′

*dros* (ChIP):

Sense: 5′-GCGTACAAGTAGTTCCCCTA-3′

Antisense: 5′-CTTGGGAACTTCGAGGAGAA-3′

*hsp27* (ChIP): as reported [26].

*hid* (ChIP): as reported [26].

Sequence data for *attA* (ChIP) and *CecA1* (ChIP) in Figure 6D were as described [44].

For real-time PCR analysis

*attA*:

Sense: 5′-GAATGGAGCTGGTCTGGATT-3′

Antisense: 5′-GCGATGACCAGAGATTAGCA-3′

*CecA1*:

Sense: 5′-TCAGTCGCTCAGACCTCACT-3′

Antisense: 5′-AGAGCGACGAAAACGAAGAT-3′

*CecA2*:

Sense: 5′-ATTAGATAGTCATCGTGGTT-3′

Antisense: 5′-GTGTTGGTCAGCACACT-3′

*def*:

Sense: 5′-TGGATCCAATTCCAGAGGAT-3′

Antisense: 5′-TGTGGTTCCAGTTCCACTTG-3′

*dip*:

Sense: 5′-GCAGCCTAACCCGAAGATTA-3′

Antisense: 5′-GAATACAGCCTCTGCACGAA-3′

*dros:*

Sense: 5′-CCGTGAGAACCTTTTCCAAT-3′

Antisense: 5′-GTATCTTCCGGACAGGCAGT-3′

*rp49* and *sqh*: Sequence data for *rp49* and *sqh* were as described [27].

For dsRNA

Primers for the synthesis of dsRNA were as described [27].
